# Letter to the editor (response to Vajro and colleagues)

**DOI:** 10.1080/21505594.2024.2316714

**Published:** 2024-02-22

**Authors:** Roger J. Grand

**Affiliations:** Institute for Cardiovascular Sciences, The Medical School, University of Birmingham, Birmingham, UK

Dear Editor,

I am most grateful to Vajro and colleagues for their generous comments concerning my recent review of human adenovirus 41 (HAdV-F41) and its possible links to severe acute hepatitis of unknown origin (AHUO) in children [1,2]. Towards the end of 2021 and in the first 6 months of 2022 over a thousand cases of hepatitis in very young children were reported worldwide [reviewed 1, 3–8]. The patients tested negative for hepatitis viruses A, B, and C (HAV, HBV, and HCV) and in many cases hepatitis viruses D and E as well as other common viruses. However, investigations showed a high incidence of human adenovirus infection and, in particular, the presence of HAdV-F41, which was surprising as it has been generally considered to infect only the gastrointestinal tract and not the liver [1,3]. Furthermore, more detailed studies on small cohorts of patients showed a very high incidence of human adeno-associated virus 2 (HAV-2) infection. Again, these were remarkable observations as adeno-associated viruses are considered to produce no clinical symptoms [1,3,6]. Over the past 12 months, the incidence of AHUO has reduced to a very low level, such that the outbreak may be considered to have ceased [9]. A final conclusion as to the cause of AHUO has not been reached, although AAV-2 is believed to have been the most likely cause, with adenovirus acting as a “helper virus” in many cases, possibly linked to an “immunity gap” caused by lockdown during the SARS-CoV-2 pandemic [1].

Vajro and colleagues have now suggested that a contributory factor to AHUO could be the presence of impure paracetamol and/or other contaminants, together with AAV-2 and HAdV-F41 [2]. Most reports of cases of AHUO make no mention of the presence of toxic chemicals, concentrating almost entirely on detected, or undetected, viruses (hepatitis viruses, adenoviruses, human herpesviruses such as Epstein–Barr virus (EBV), human immunodeficiency virus (HIV), cytomegalovirus (CMV), and human papilloma virus (HPV)). Interestingly, in an early study, it was suggested that the presence of some form of “toxin” or “environmental factors” were being considered as contributory causes of hepatitis, although no details were given [10]. However, in a retrospective analysis of AHUO cases in the UK occurring in early 2022 it was concluded that “no common toxin exposure” had occurred although, again, it was not clear what tests had been performed [11]. Similarly, in a technical report from the Center for Disease Control it was stated that “although epidemiological investigations are still underway for the majority of PUIs (patients under investigation), no associations have been found with pets, food, medication, toxins, or other exposures” [9]. In several reports, published in 2022, it was suggested that attention should be paid to the possibility of the involvement of toxic chemicals in AHUO. For example, in the WHO release, it was recommended that the investigation of “other potential explanatory/contributing factors (either other infection, toxins, or medications)” as well as viruses be undertaken [12]. Obviously, clinicians have been aware of the possibility that toxic chemicals, such as impure paracetamol, could have contributed to AHUO but as little or no information is available on specific tests which may have been performed, or should be performed, it is difficult to say more than to heed the warnings given that such a possibility be borne in mind in the future, as recommended by Vajro and colleagues [2].
